# CDDO-Me Attenuates Astroglial Autophagy via Nrf2-, ERK1/2-SP1- and Src-CK2-PTEN-PI3K/AKT-Mediated Signaling Pathways in the Hippocampus of Chronic Epilepsy Rats

**DOI:** 10.3390/antiox10050655

**Published:** 2021-04-23

**Authors:** Ji-Eun Kim, Tae-Cheon Kang

**Affiliations:** 1 Department of Anatomy and Neurobiology, College of Medicine, Hallym University, Chuncheon 24252, Korea; 2 Institute of Epilepsy Research, College of Medicine, Hallym University, Chuncheon 24252, Korea

**Keywords:** astrocyte, Bif-1, clasmatodendrosis, GSK3β, HSP25, seizure

## Abstract

Clasmatodendrosis is an autophagic astroglial death showing extensive swollen cell bodies with vacuoles and disintegrated/beaded processes. This astroglial degeneration is closely relevant to the synchronous epileptiform discharges. However, the underlying molecular mechanisms and the roles of clasmatodendrosis in spontaneous seizure activity are still unknown. The 2-cyano-3,12-dioxo-oleana-1,9(11)-dien-28-oic acid methyl ester (CDDO-Me; RTA 402) is one of the activators for nuclear factor-erythroid 2-related factor 2 (Nrf2) that is a redox-sensitive transcription factor. In the present study, we explored the effects of CDDO-Me on clasmatodendrosis in chronic epilepsy rats, which could prevent epilepsy-related complications. In the present study, clasmatodendritic astrocytes showed reduced Nrf2 expression and its nuclear accumulation, which were restored by CDDO-Me. CDDO-Me also abrogated heat shock protein 25 (HSP25) upregulation in clasmatodendritic astrocytes by regulating extracellular signal-related kinases 1/2 (ERK1/2)-specificity protein 1 (SP1)- and Src-casein kinase 2 (CK2)-phosphatase and tensin homolog deleted on chromosome 10 (PTEN)-phosphatidylinositol-3-kinase (PI3K)-AKT-glycogen synthase kinase 3β (GSK3β)-bax-interacting factor 1 (Bif-1)-mediated signaling pathways in chronic epilepsy rats. In addition, CDDO-Me ameliorated spontaneous seizure duration, but not seizure frequency and behavioral seizure severity. Therefore, our findings suggest that clasmatodendrosis may affect seizure duration in chronic epilepsy rats, and that CDDO-Me may attenuate autophagic astroglial degeneration by regulating various signaling pathways.

## 1. Introduction

Epilepsy is one of the most common chronic neurological diseases. The mean prevalence rate of active epilepsy is 8% worldwide. The etiology of epilepsy is unknown (idiopathic) or related to disease states including brain tumors and traumatic injury [1,2]. The main symptom of epilepsy is the presence of spontaneous episodes of abnormal excessive neuronal discharges. This seizure activity results in neuronal loss in the various brain regions, especially in the hippocampus (hippocampal sclerosis) [3,4]. Similar to other brain injuries, neuronal loss and synaptic rearrangement induce astroglial activation (reactive astrogliosis), which may contribute to epileptogenesis [5,6,7].

Astrocytes are key players in the regulation of extracellular glutamate concentration, ion homeostasis and neuronal functionality, and are believed to be resistant to harmful stresses [8,9]. More than 100 years ago, however, Alzheimer reported irreversible astroglial injury characterized by extensive swollen cell bodies with vacuoles and disintegrated/beaded processes, and Cajal termed it as “clasmatodendrosis” [10]. In addition, Revuelta et al. [11] reported terminal deoxynucleotidyl transferase dUTP nick-end labeling (TUNEL)-negative astroglial degeneration in the CA1 region and the amygdalar complex after kainic acid administration. We have also reported clasmatodendrosis in the CA1 region of chronic epilepsy rats, although TUNEL-positive astroglial death in the molecular layer (not the hilus) of the dentate gyrus was observed in the acute stage following status epilepticus (SE, a continuous unremitting seizure activity) [7,12,13,14,15,16]. Since clasmatodendritic astrocytes show eosinophilic cytoplasm with vacuoles, at first we reported that clasmatodendrosis might be coagulative necrotic events and be one of the epilepsy-related complications [16,17]. Later, we fortunately found that vacuoles in clasmatodendritic astrocytes are active lysosomes, which are required for the essential activation of autophagy [12,13]. Thereafter, ultrastructural studies by other investigators confirmed that clasmatodendritic astrocytes showed autophagocytosis and ubiquitin proteasome system (UPS)-mediated astroglial degeneration under various pathological conditions [18,19]. Thus, clasmatodendrosis is an autophagic astroglial degeneration. This is because aberrant regulation of autophagy results in non-apoptotic programmed cell death (type II programmed cell death) independent of caspase activity [20,21,22]. Furthermore, clasmatodendrosis is closely relevant to the synchronous epileptiform discharges [16]. However, the underlying molecular mechanisms of clasmatodendrosis and the therapeutic strategies to protect astrocytes from this irreversible degeneration are still elusive, although extracellular signal-related kinases 1/2 (ERK1/2)-specificity protein 1 (SP1)-mediated prolonged heat shock protein 25 (HSP25) can lead to clasmatodendrosis [23,24,25].

Nuclear factor-erythroid 2-related factor 2 (Nrf2) is involved in the maintenance of redox homeostasis by regulating antioxidant-response element (ARE)-dependent transcription [26,27]. Under physiological conditions, Nrf2 is bound to Kelch-like erythroid cell-derived protein with CNC homology (ECH)-associated protein 1 (Keap1), and is subsequently ubiquitinated through the cullin-3 (Cul3)-based E3 ubiquitin ligase complex. Under oxidative stress, the oxidation of SH-groups in Keap1 liberates Nrf2, and facilitates nuclear Nrf2 translocation, which induces ARE-mediated transactivation of redox enzymes, including glutathione S-transferases (GSTs) and scavengers of reactive oxygen species (ROS). Glycogen synthase kinase 3β (GSK3β) also inhibits nuclear Nrf2 translocation by phosphorylation [28,29,30,31]. Furthermore, Nrf2 inhibits SP1 activation, which is one of the key molecules inducing clasmatodendrosis [25,32]. Indeed, Nrf2 protects neurons and astrocytes from SE [33,34,35,36]. Thus, it is noteworthy to explore whether 2-cyano-3,12-dioxo-oleana-1,9(11)-dien-28-oic acid methyl ester (CDDO-Me; RTA 402, a Nrf2 activator) affects clasmatodendrosis and spontaneous seizure activity in chronic epilepsy rats.

Here, we demonstrate that CDDO-Me attenuated HSP25-induced clasmatodendrosis through Nrf2-, ERK1/2-SP1- and Src-casein kinase 2 (CK2)-phosphatase and tensin homolog deleted on chromosome 10 (PTEN)-phosphatidylinositol-3-kinase (PI3K)-AKT-GSK3β-bax-interacting factor 1 (Bif-1)-mediated signaling pathways in chronic epilepsy rats. In addition, CDDO-Me ameliorated seizure duration, but not seizure frequency or behavioral seizure severity. Therefore, our findings suggest that clasmatodendrosis may increase seizure duration in chronic epilepsy rats, and CDDO-Me may attenuate this autophagic astroglial degeneration via various signaling pathways.

## 2. Materials and Methods

### 2.1. Experimental Animals and Chemicals

Adult male Sprague-Dawley (SD) rats (7 weeks old) were used in the present study. Animals were kept under controlled light and environmental conditions (22 ± 22 °C, humidity 55 ± 5%, a light-dark cycle on a 12-h on-off cycle) with ad libitum access to water and food throughout the experiments. All experimental protocols were approved by the Institutional Animal Care and Use Committee of Hallym University (code number: Hallym 2018-2, approval date: 26 April 2018). All reagents were obtained from Sigma-Aldrich (St. Louis, MO, USA), except as noted [14,15].

### 2.2. Epilepsy Model

Animals were subjected to the LiCl-pilocarpine model of temporal lobe epilepsy (TLE). Rats were given LiCl (127 mg/kg, i.p.) 24 h before the pilocarpine treatment. Animals were treated with pilocarpine (30 mg/kg, i.p.) 20 min after atropine methylbromide (5 mg/kg i.p.). Two hours after SE onset, diazepam (Valium; Hoffmann-la Roche, Neuilly-sur-Seine, France; 10 mg/kg, i.p.) was administered to terminate SE and repeated, as needed. Control animals received saline in place of pilocarpine [14,15,16,37,38]. Animals were video-monitored 8 h a day for selecting chronic epileptic rats showing spontaneous recurrent seizures. Behavioral seizure severity was evaluated according to Racine’s scale [37,38].

### 2.3. Electrode Implantation, CDDO-Me Trials and Quantification of Seizure Activity

Control and epileptic rats were implanted with a monopolar stainless steel electrode (Plastics One, Roanoke, VA, United States) in the right hippocampus under Isoflurane anesthesia (3% induction, 1.5–2% for surgery and 1.5% maintenance in a 65:35 mixture of N_2_O:O_2_) using the following coordinates: −3.8 mm posterior; 2.0 mm lateral; −2.6 mm depth. Animals were also implanted with a brain infusion kit 1 and an Alzet 1007D osmotic pump (Alzet, Cupertino, CA, USA) to infuse with vehicle or CDDO-Me (10 μM) into the right lateral ventricle [35,36]. The correct location of infusion needle into the ventricle was confirmed during brain sections and when sampling tissues for Western blot. Electrode and infusion needle were secured to the exposed skull with dental acrylic. Three days after surgery, an electroencephalogram (EEG) was recorded 2 h a day at the same time over 4 days [37,38,39]. Behavioral seizure severity was also evaluated as aforementioned [37,38]. After recording, animals were used for Western blot and immunofluorescent study.

### 2.4. Western Blots

Animals were sacrificed via decapitation. The brains were quickly removed and coronally cut to 1 mm thickness (approximately 3–4 mm posterior to the bregma) using rodent brain matrix (World Precision Instruments, Sarasota, FL, United States) on ice. Thereafter, the stratum radiatum of the CA1 region of the dorsal hippocampus were rapidly dissected out in cold (4 °C) artificial cerebrospinal fluid under stereomicroscope [14]. The CA1 tissues were homogenized and protein concentration determined using a Micro BCA Protein Assay Kit (Pierce Chemical, Rockford, IL, United States). Western blot was performed by the standard protocol as follows: following electrophoresis, proteins were transferred to nitrocellulose membranes. Membranes were incubated overnight at 4 °C with 2% bovine serum albumin (BSA) in Tris-buffered saline (TBS; in 10 mM Tris, 150 NaCl, pH 7.5 and 0.05% Tween 20) and then in primary antibodies (Supplementary Table S1). Subsequently, the membranes were incubated for 1 h at room temperature in a solution containing horseradish peroxidase (HRP)-conjugated secondary antibodies. A chemiluminescence signal was detected by luminol substrate reaction (ECL Western Blotting System, GE Healthcare Korea, Seoul, Korea). The values of each sample were calculated with the corresponding amount of β-actin. The ratio of phosphoprotein to total protein was described as phosphorylation level [25,37,38,39].

### 2.5. Immunohistochemistry, Cell Counts and Measurement of Fluorescent Intensity

Under urethane anesthesia (1.5 g/kg, i.p.), animals were transcardially and subsequently perfused with 0.9% saline followed by 4% paraformaldehyde in 0.1 M phosphate buffer (PB, pH 7.4). The brains were isolated and post-fixed in the same fixative overnight, and then stored in 30% sucrose/0.1 M PBS. Coronal sections were sliced at a 30-μm thickness with a freezing microtome. Then, sections were reacted with in 0.1% bovine serum albumin and successively primary antibody (Supplementary Table S1). After washing, sections were further incubated in appropriate Cy2- and Cy3-conjugated secondary antibodies. Immunofluorescence was observed using an AxioScope microscope (Carl Zeiss Korea, Seoul, Korea). To establish the specificity of the immunostaining, a negative control test was carried out with normal mouse serum (#31880, ThermoFisher Korea, Seoul, Korea), normal rabbit serum (#31883, ThermoFisher Korea, Seoul, Korea), mouse IgG1 isotype control (#02-6100, ThermoFisher Korea, Seoul, Korea) and mouse IgG2a isotype control (#02-6200, ThermoFisher Korea, Seoul, Korea), instead of the primary antibodies. No immunoreactivity was observed for the negative control in any structures (Figure S1) [14,15]. All experimental procedures in this study were performed under the same conditions and in parallel. For cell counts, sections (10 sections per each animal) were captured and areas of interest (1 × 10^4^ μm^2^) were selected from the CA1 region using an AxioImage M2 microscope. Thereafter, cell counts were performed using AxioVision Rel. 4.8 Software. For measurement of fluorescent intensity, 30 areas/rat (400 μm^2^/area) were randomly selected within the stratum radiatum of the CA1 region (15 sections from each animal, *n* = 7 in each group). After capture, green or red channel was converted to a grayscale image, and mean intensity was measured using AxioVision Rel. 4.8 software (Carl Zeiss Korea, Seoul, Korea). Fluorescent intensity was normalized by setting the mean background. In addition, colocalization of Nrf2 with 4′,6-diamidino-2-phenylindole (DAPI) was analyzed for nuclear Nrf2 intensity. Thereafter, the ratio of nuclear:cytosolic Nrf2 intensity was calculated. Cell counts and measurement of fluorescent intensities were performed by two different investigators who were blind to the classification of tissues [25,37,38,39].

### 2.6. Data Analysis

Data were analyzed using Student *t*-test or one-way analysis of variance (ANOVA) followed by Bonferroni’s post hoc comparisons after evaluating the values on normality using Shapiro–Wilk *W*-test. Mann–Whitney U-test was also used to determine statistical significance of data. A *p*-value less than 0.05 was considered to be significant [25,37,38,39].

## 3. Results

### 3.1. The Nrf2 Protein Level and Its Nuclear Translocation Are Reduced in Clasmatodendritic Astrocytes

**Figure 1:**
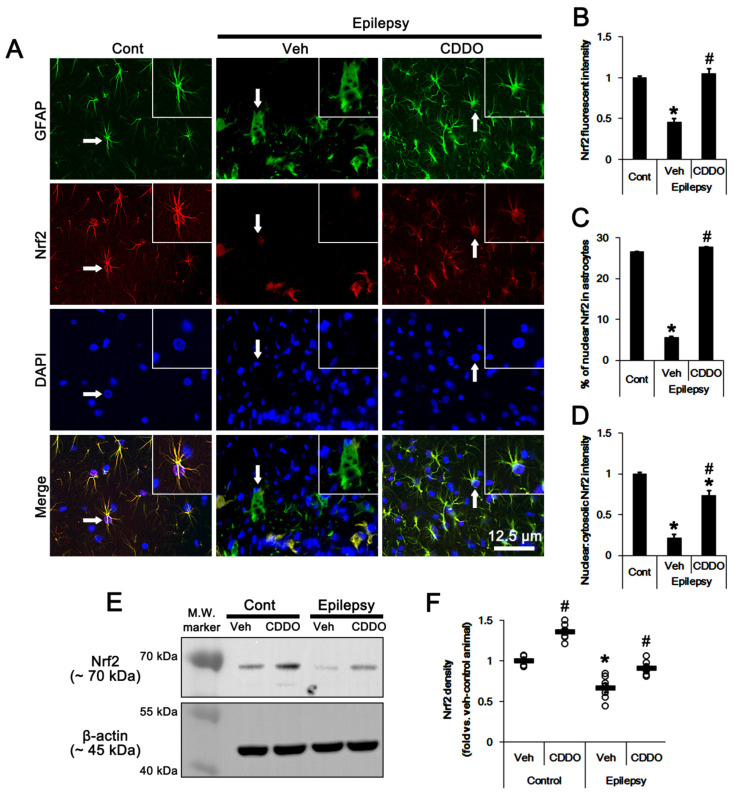
**Effects of CDDO-Me on nuclear factor-erythroid 2-related factor 2 (Nrf2) expression and its nuclear accumulation in CA1 astrocytes of control and epileptic rats.** As compared to control animals (Cont), Nrf2 expression and its nuclear accumulation were reduced in CA1 astrocytes in epileptic rats. CDDO-Me increased Nrf2 expression and its nuclear accumulation in CA1 astrocytes, as compared to vehicle (Veh). (**A**) Representative photos demonstrating astroglial Nrf2 expression in CA1 astrocytes. Arrows indicate the inserted magnification photos. (**B**–**D**) Quantifications of Nrf2 expression (**B**), the fraction of astrocytes showing Nrf2 signals in total astrocytes (**C**) and the ratio of nuclear:cytosolic Nrf2 intensity (**D**) in CA1 astrocytes. Error bars indicate SEM (*,# *p* < 0.05 vs. control and vehicle-treated epileptic rats, respectively; *n* = 7). (**E**) Representative Western blot for Nrf2 in the CA1 region. (**F**) Quantitative values (mean ± SEM) of the Western blot data concerning Nrf2 expression level (*n* = 7, respectively). Open circles indicate each value. Horizontal bars indicate the mean value. Significant differences are *,# *p* < 0.05 vs. control animals and vehicle-treated animals.

In control animals, Nrf2 expression was mainly observed in the cytoplasm of astrocytes in the stratum radiatum in the CA1 region (referred to as CA1 astrocytes below). Some CA1 astrocytes (~27%) also demonstrated nuclear Nrf2 signals (Figure 1A–D). In epileptic rats, Nrf2 expression was reduced in clasmatodendritic CA1 astrocytes that had round-shaped edematous cell bodies, vacuoles, loss of distal processes and glial fibrillary acidic protein (GFAP) tangles (*t*_(12)_ = 12.6, *p* < 0.00001 vs. control animals, Student *t*-test, *n* = 7, respectively; Figure 1A,B). Only a few CA1 astrocytes (~ 6%) showed nuclear Nrf2 signals (*t*_(12)_ = 9.6, *p* < 0.00001 vs. control animals, Student *t*-test, *n* = 7, respectively; Figure 1A,C). Western blots also revealed that Nrf2 level was reduced to ~0.67-fold of control animal level in epileptic rats. CDDO-Me increased Nrf2 level to ~1.36- and ~1.35-fold of vehicle level in control and epileptic animals, respectively (*F*_(3,24)_ = 56.7, *p* < 0.00001, one-way ANOVA, *n* = 7, respectively; Figure 1E,F, Figure S2). In addition, CDDO-Me attenuated clasmatodendritic changes in CA1 astrocytes of epileptic animals, accompanied by elevated Nrf2 fluorescent intensity (*F*_(2,18)_ = 68.1, *p* < 0.00001, one-way ANOVA, *n* = 7, respectively; Figure 1A,B) and nuclear Nrf2 accumulation (*F*_(2,18)_ = 1081.1, *p* < 0.00001, one-way ANOVA, *n* = 7, respectively; Figure 1A,B). Thus, CDDO-Me also increased the ratio of nuclear:cytosolic intensity in CA1 astrocytes (*F*_(2,18)_ = 19.3, *p* = 0.00003, one-way ANOVA, *n* = 7, respectively; Figure 1A,D). These findings indicate that the reduced Nrf2 level may be involved in autophagic CA1 astroglial degeneration in epileptic animals.

### 3.2. CDDO-Me Regulates ERK1/2-Mediated HSP25 Induction in CA1 Astrocytes

**Figure 2:**
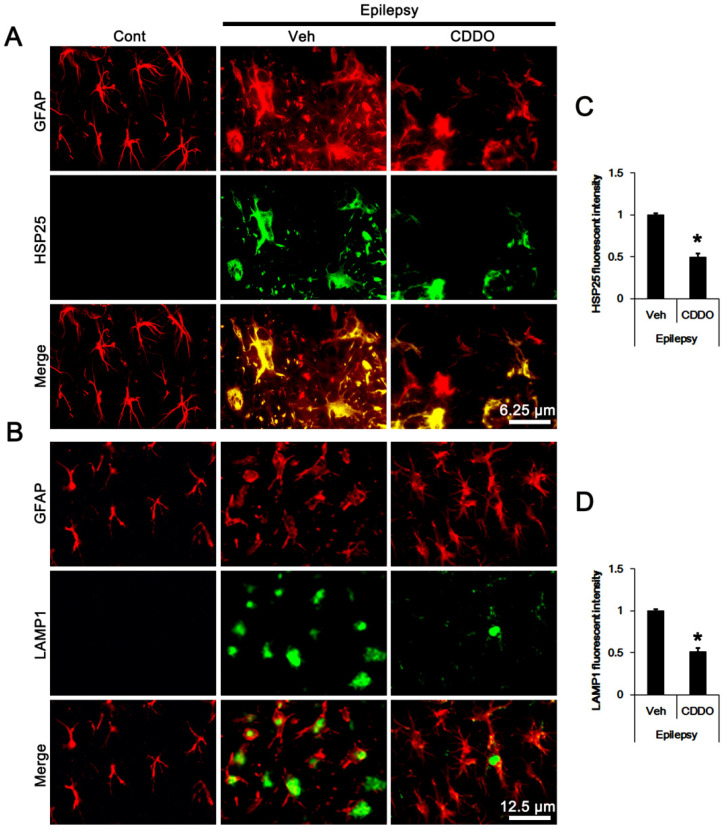
**Effects of CDDO-Me on HSP25 and LAMP1 expressions in CA1 astrocytes in control and epileptic rats.** As compared to control animals (Cont), expressions of heat shock protein 25 (HSP25) and lysosomal-associated membrane protein 1 (LAMP1) were increased in CA1 astrocytes in epileptic rats. CDDO-Me decreased both HSP25 and LAMP1 expressions in CA1 astrocytes, as compared to vehicle (Veh). (**A**,**B**) Representative photos demonstrating astroglial HSP25 (**A**) and LAMP1 (**B**) expressions in CA1 astrocytes. (**C**,**D**) Quantifications of HSP25 (**C**) and LAMP1 (**D**) expression in CA1 astrocytes. Error bars indicate SEM (* *p* < 0.05 vs. vehicle-treated epileptic rats, respectively; *n* = 7).

In previous studies [24,25], we reported that ERK1/2-mediated prolonged HSP25 upregulation lead to astroglial autophagy. Thus, we investigated whether CDDO-Me affected clasmatodendrosis by influencing sustained HSP25 expression in the epileptic hippocampus. In control animals, HSP25- and lysosomal-associated membrane protein 1 (LAMP1)-positive astrocytes were rarely observed in the hippocampus (Figure 2A,B). In epileptic rats, HSP25-positive astrocytes demonstrated the clasmatodendritic changes with LAMP1 positive vacuoles (Figure 2A,B). In CDDO-Me-treated epileptic rats, CA1 astrocytes showed typical reactive astrogliosis rather than clasmatodendrosis. CDDO-Me reduced the fluorescent intensities of HSP25 (*t*_(12)_ = 6.3, *p* = 0.00004 vs. vehicle, Student *t*-test, *n* = 7, respectively; Figure 2A,C) and LAMP1 (*t*_(12)_ = 6.8, *p* = 0.00002 vs. vehicle, Student *t*-test, *n* = 7, respectively; Figure 2B,D) in CA1 astrocytes, respectively.

**Figure 3:**
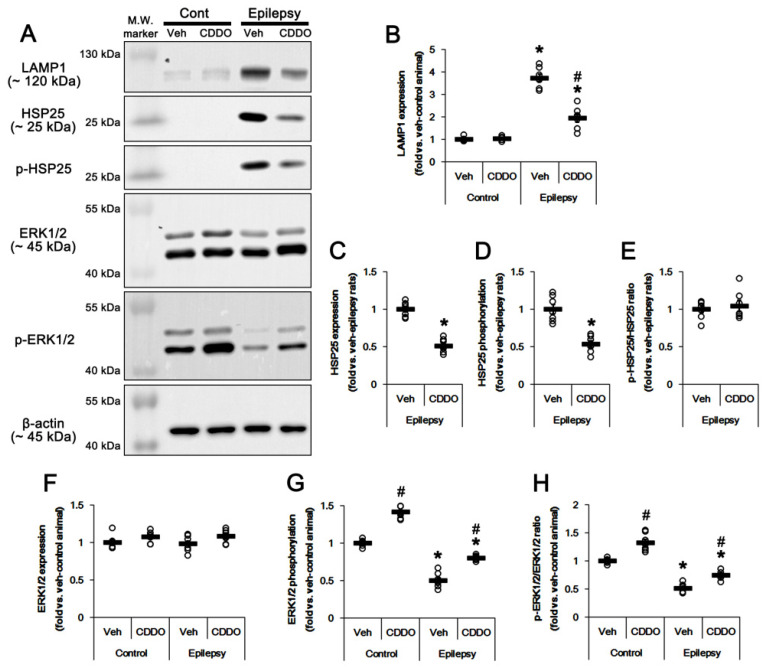
**Effects of CDDO-Me on LAMP1, HSP25, p-HSP25, ERK1/2 and p-ERK1/2 levels in the CA1 region of control and epileptic rats.** As compared to control animals (Cont), LAMP1, HSP25 and p-HSP25 levels were enhanced in the CA1 region of epileptic rats. However, extracellular signal-related kinases 1/2 (ERK1/2) phosphorylation (p-ERK1/2) level was diminished in epileptic rats without altering its protein level. CDDO-Me treatment decreased protein expression and phosphorylation of LAMP1 and HSP25, but increased ERK1/2 phosphorylation. (**A**) Representative Western blot for LAMP1, HSP25, p-HSP25, ERK1/2 and p-ERK1/2 in the CA1 regions. (**B**–**H**) Quantitative values (mean ± SEM) of protein, phosphorylation and phosphorylation ratio concerning LAMP1 (**B**), HSP25 (**C**–**E**) and ERK1/2 (**F**–**H**) (*n* = 7). Open circles indicate each value. Horizontal bars indicate the mean value. Significant differences are *,# *p* < 0.05 vs. control animals and vehicle-treated animals.

Western blots showed the increase in LAMP1 expression in the epileptic hippocampus (*t*_(12)_ = 16.1, *p* < 0.00001 vs. control animals, Student *t*-test, *n* = 7, respectively; Figure 3A,B), accompanied by upregulated HSP25 and phospho (p)-HSP25 levels (Figure 3A). In contrast, p-ERK1/2 level was significantly lower in the epileptic hippocampus than the control (*t*_(12)_ = 12.1, *p* < 0.00001 vs. control animals, Student *t*-test, *n* = 7, respectively; Figure 3A,F–H, Figure S2). CDDO-Me decreased LAMP1 expression level to ~0.52-fold of vehicle level in epileptic animals, respectively (*F*_(3,24)_ = 106, *p* < 0.00001 vs. vehicle, one-way ANOVA, *n* = 7, respectively; Figure 3A,B). CDDO-Me also reduced HSP25 (*t*_(12)_ = 9.3, *p* < 0.00001 vs. vehicle, Student *t*-test, *n* = 7, respectively; Figure 3A,C) and p-HSP25 levels (*t*_(12)_ = 6.1, *p* = 0.00006 vs. vehicle, Student *t*-test, *n* = 7, respectively; Figure 3A,D) without altering the p-HSP25 phosphorylation ratio (Figure 3A,E). However, CDDO-Me increased p-ERK1/2 level (*F*_(3,24)_ = 211.1, *p* < 0.00001 vs. vehicle, one-way ANOVA, *n* = 7, respectively; Figure 3A,G) and p-ERK1/2 ratio (*F*_(3,24)_ = 88.6, *p* < 0.00001 vs. vehicle, one-way ANOVA, *n* = 7, respectively; Figure 3A,H) in control and epileptic rats without changing ERK1/2 expression level (Figure 3A,F, Figure S2). Considering CDDO-Me induced ERK1/2 activation, and the negative effect of ERK1/2 on SP1-mediated HSP25 induction [25,35], these findings indicate that CDDO-ME may ameliorate clasmatodendrosis of CA1 astrocytes by activating ERK1/2-mediated HSP25 inhibition.

### 3.3. CDDO-Me Leads to PTEN-Mediated AKT Inhibition in the Epileptic Hippocampus

**Figure 4:**
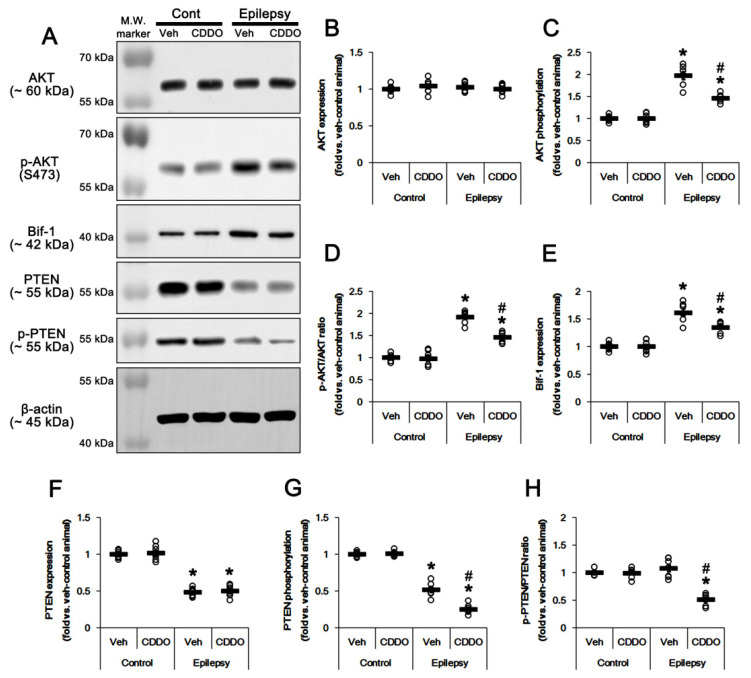
**Effects of CDDO-Me on AKT, p-AKT, Bif-1, PTEN and p-PTEN levels in the CA1 region of control and epileptic rats.** As compared to control animals (Cont), AKT-S473 phosphorylation and bax-interacting factor 1 (Bif-1) expression were lower in the CA1 region of epileptic rats. However, PTEN expression and its phosphorylation were also decreased in epileptic rats. CDDO-Me treatment attenuated the increases in AKT phosphorylation and Bif-1 expression in epileptic rats. In addition, CDDO-Me reduced p-PTEN level in epileptic rats without affecting its protein expression level. (**A**) Representative Western blot for AKT, p-AKT, Bif-1, PTEN and p-PTEN in the CA1 regions. (**B**–**H**) Quantitative values (mean ± SEM) of protein, phosphorylation and phosphorylation ratio concerning AKT (**B**–**D**), Bif-1 (**E**) and PTEN (**F**–**H**) (*n* = 7, respectively). Open circles indicate each value. Horizontal bars indicate the mean value. Significant differences are *,# *p* < 0.05 vs. control animals and vehicle-treated animals.

Sustained HSP25 induction activates AKT serine (S) 473/Bif-1-mediated autophagy in CA1 astrocytes, and AKT inhibition attenuates clasmatodendrosis [25]. Thus, it is likely that CDDO-Me may also ameliorate clasmatodendrosis in CA1 astrocytes by inhibiting AKT activity. In the present study, p-AKT level (*t*_(12)_ = 10.7, *p* < 0.00001 vs. control animals, Student *t*-test, *n* = 7, respectively; Figure 4A–C), p-AKT ratio (*t*_(12)_ = 15.3, *p* < 0.00001 vs. control animals, Student *t*-test, *n* = 7, respectively; Figure 4A,D, Figure S3) and Bif-1 expression (*t*_(12)_ = 8.8, *p* < 0.00001 vs. control animals, Student *t*-test, *n* = 7, respectively; Figure 4A,E, Figure S3) in the epileptic hippocampus was higher than that in the control hippocampus. CDDO-Me attenuated the elevated p-AKT level (*F*_(3,24)_ = 76.6, *p* < 0.00001 vs. vehicle, one-way ANOVA, *n* = 7, respectively; Figure 4A,C, Figure S3), p-AKT ratio (*F*_(3,24)_ = 79.4, *p* < 0.00001 vs. vehicle, one-way ANOVA, *n* = 7, respectively; Figure 4A,D) and Bif-1 level (*F*_(3,24)_ = 44.4, *p* < 0.00001 vs. vehicle, one-way ANOVA, *n* = 7, respectively; Figure 4A,E). These findings indicate that CDDO-Me may inhibit AKT/Bif-1-mediated astroglial autophagy in the epileptic hippocampus.

Since PTEN negatively regulates AKT phosphorylation [40], we also validated the effect of CDDO-Me on PTEN expression and its phosphorylation. Consistent with previous studies [41,42], PTEN protein level (*t*_(12)_ = 17.4, *p* < 0.00001 vs. control animals, Student *t*-test, *n* = 7, respectively; Figure 4A,F, Figure S3) and its phosphorylation levels were downregulated in the epileptic hippocampus (*t*_(12)_ = 12.9, *p* < 0.00001 vs. control animals, Student *t*-test, *n* = 7, respectively; Figure 4A,G, Figure S3). However, p-PTEN ratio was unaltered (Figure 4A,H). Considering that PTEN phosphorylation represents its inactivation [43], these findings indicate that the reduced PTEN expression, but not its post-modification (phosphorylation), may decrease PTEN activity in the epileptic hippocampus. CDDO-Me reduced p-PTEN level (*F*_(3,24)_ = 253.9, *p* < 0.00001 vs. vehicle, one-way ANOVA, *n* = 7, respectively; Figure 4A,G) and its ratio (*F*_(3,24)_ = 37.4, *p* < 0.00001 vs. vehicle, one-way ANOVA, *n* = 7, respectively; Figure 4A,H) in the epileptic hippocampus without affecting PTEN protein level (Figure 4A,F). Thus, it is likely that CDDO-Me may increase PTEN activity in the epileptic hippocampus by reducing PTEN phosphorylation. These findings represent that CDDO-Me may abrogate clasmatodendrosis by facilitating PTEN-mediated AKT inhibition in the epileptic hippocampus.

### 3.4. CDDO-Me Inhibits CK2 Phosphorylation in the Epileptic Hippocampus

**Figure 5:**
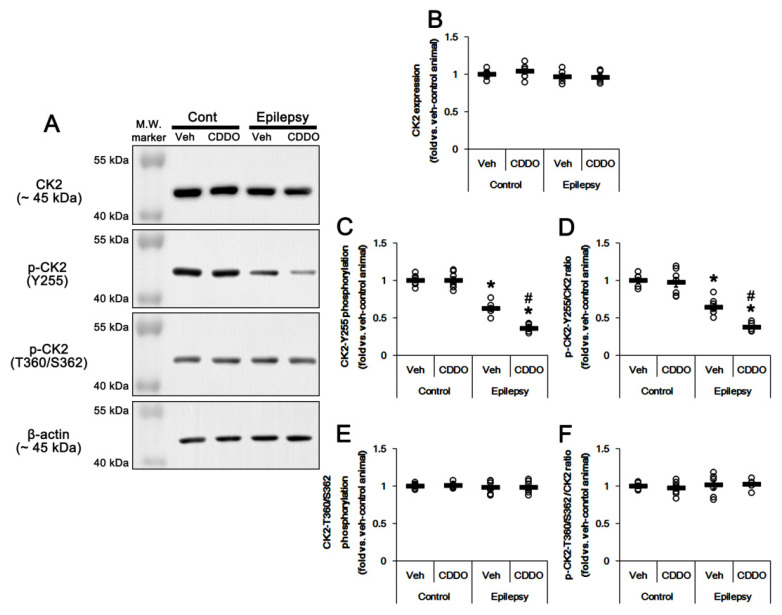
**Effects of CDDO-Me on CK2 and p-CK2 levels in the CA1 region of control and epileptic rats.** As compared to control animals (Cont), casein kinase 2 (CK2)-Y255, but not T360/S362, phosphorylation was reduced in the CA1 region of epileptic rats without altering its expression level. CDDO-Me treatment decreased CK2-Y255 phosphorylation more in epileptic rats. (**A**) Representative Western blot for CK2 and p-CK2 in the CA1 regions. (**B**–**F**) Quantitative values (mean ± SEM) of protein, phosphorylation and phosphorylation ratio concerning CK2 (**B**), p-CK2-Y255 (**C**,**D**) and p-CK2-T360/S362 (**E**,**F**) (*n* = 7). Open circles indicate each value. Horizontal bars indicate the mean value. Significant differences are *,# *p* < 0.05 vs. control animals and vehicle-treated animals.

CK2 was the first to be identified and is the most prominently characterized PTEN kinase. CK2 regulates PTEN stability, activity and subcellular localization via phosphorylation of PTEN at S370, S380, threonine (T) 382, T383 and S385 sites [44,45]. CK2 activity is regulated by the phosphorylation of tyrosine (Y) 255 and T360/S362 sites, which are phosphorylated by Src family protein tyrosine kinases and ERK1/2, respectively [46,47]. Thus, we explored whether CDDO-Me affected CK2 expression and its phosphorylation in the epileptic hippocampus. Consistent with a previous study demonstrating the unchanged CK2 expression after kainic acid injection [37,48], CK2 expression was similarly observed in the epileptic hippocampus, as compared to the control (Figure 5A,B, Figure S4). However, CK2 Y255 phosphorylation (*t*_(12)_ = 8.93, *p* **<** 0.00001 vs. control animals, Student *t*-test, *n* = 7, respectively; Figure 5A,C) and its ratio (*t*_(12)_ = 6.73, *p* = 0.00002 vs. control animals, Student *t*-test, *n* = 7, respectively; Figure 5A,D, Figure S4) were reduced in the epileptic hippocampus. CK2 T360/S362 phosphorylation level and its ratio were similar to those observed in control animals (Figure 5A,E,F, Figure S4). Although CDDO-Me did not affect CK2 phosphorylation in the normal hippocampus, it alleviated CK2 Y255 phosphorylation level (*F*_(3,24)_ = 101.7, *p* < 0.00001 vs. vehicle, one-way ANOVA, *n* = 7, respectively; Figure 5A,C) and its ratio (*F*_(3,24)_ = 47.1, *p* < 0.00001 vs. vehicle, one-way ANOVA, *n* = 7, respectively; Figure 5A,D, Figure S4) without altering T360/S362 phosphorylation (Figure 5A,E,F). Thus, these findings indicate that CDDO-Me may activate PTEN activity via inhibiting CK2 Y255 phosphorylation in the epileptic hippocampus.

### 3.5. CDDO-Me Inhibits Src Family Phosphorylation at Y416 Site in the Epileptic Hippocampus

**Figure 6:**
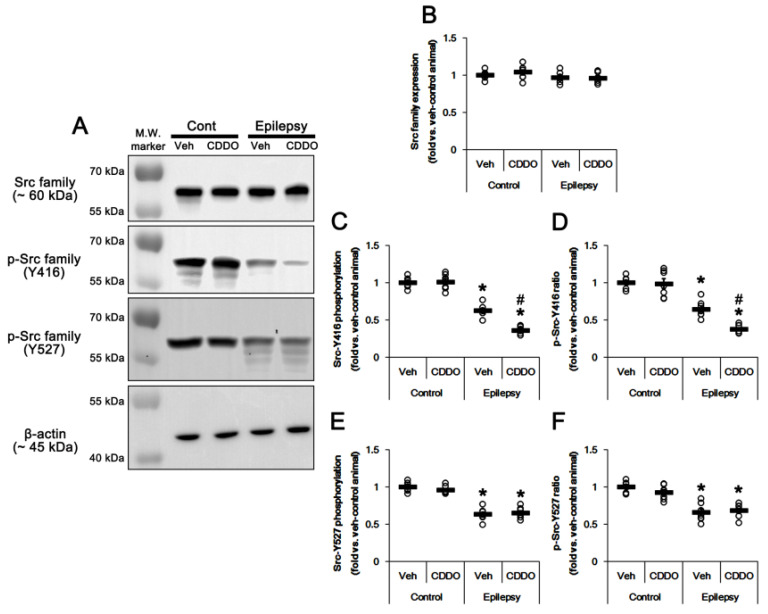
**Effects of CDDO-Me on Src and p-Src levels in the CA1 region of control and epileptic rats.** As compared to control animals (Cont), Src-Y416 and Y527 phosphorylation was reduced in the CA1 region of epileptic rats without altering its expression level. CDDO-Me treatment decreased Src-Y416, but not Y527, phosphorylation in epileptic rats. (**A**) Representative Western blot for Src and p-Src in the CA1 regions. (**B**–**F**) Quantitative values (mean ± SEM) of protein, phosphorylation and phosphorylation ratio concerning Src (*n* = 7, respectively). Open circles indicate each value. Horizontal bars indicate the mean value. Significant differences are *,# *p* < 0.05 vs. control animals and vehicle-treated animals.

As aforementioned, CK2 increased its catalytic activity by Src family protein tyrosine kinases-mediated phosphorylation at the Y255 residue [37,46]. The Src family of protein tyrosine kinase activities are regulated by tyrosine phosphorylation at two sites, but with opposing effects. Autophosphorylation of tyrosine (Y) 396 (equivalent to Y416 of Src), located in the catalytic domain, upregulates Src kinase activity. Y507 phosphorylation (equivalent to Y527 of Src) inactivates the kinase activity, while dephosphorylation of this site is not sufficient for full kinase activation [49,50]. Interestingly, CDDO-Me affects Src and AKT activation [51,52]. Thus, we evaluated the effect of CDDO-Me on Src family activities. As compared to the control hippocampus, Src family-Y416 phosphorylation was reduced in the epileptic hippocampus without changing Src family expression (*t*_(12)_ = 19.4, *p* < 0.00001 vs. control animals, Student *t*-test, *n* = 7, respectively; Figure 6A–C, Figure S5). p-Src-Y416 family ratio was also decreased in the epileptic hippocampus (*t*_(12)_ = 6.7, *p* = 0.00002 vs. control animals, Student *t*-test, *n* = 7, respectively; Figure 6A,D, Figure S5). CDDO-Me reduced the p-Src-Y416 level (*F*_(3,24)_ = 108.3, *p* < 0.00001 vs. vehicle, one-way ANOVA, *n* = 7, respectively; Figure 6A,C, Figure S5) and its ratio (*F*_(3,24)_ = 46.7, *p* < 0.00001 vs. vehicle, one-way ANOVA, *n* = 7, respectively; Figure 6A,D, Figure S5) in the epileptic hippocampus, but not the normal hippocampus. In addition, Src family-Y527 phosphorylation was decreased in the epileptic hippocampus (*t*_(12)_ = 9.1, *p* < 0.00001 vs. control animals, Student *t*-test, *n* = 7, respectively; Figure 6A,E) and p-Src-Y527 ratio (*t*_(12)_ = 6.5, *p* = 0.00003 vs. control animals, Student *t*-test, *n* = 7, respectively; Figure 6A,F). However, CDDO-Me did not affect p-Src-Y527 level (*t*_(12)_ = 0.5, *p* = 0.65 vs. vehicle, Student *t*-test, *n* = 7, respectively; Figure 6A,E, Figure S5) and its ratio (*t*_(12)_ = 0.4, *p* = 0.68 vs. vehicle, Student *t*-test, *n* = 7, respectively; Figure 6A,F) in the epileptic hippocampus. These findings indicate that CDDO-Me may activate PTEN by inhibiting the Src family-CK2 signaling pathway.

### 3.6. CDDO-Me Decreases Seizure Duration, But Not Seizure Frequency or Its Intensity

**Figure 7:**
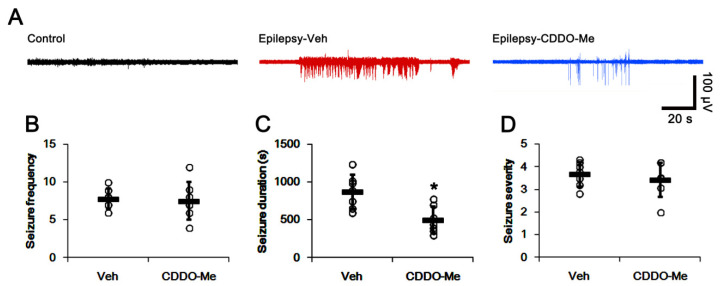
**Effects of CDDO-Me on spontaneous seizure activity in epileptic rats.** CDDO-Me treatment reduces seizure duration, but not seizure frequency and its severity in epileptic rats. (**A**) Representative EEG traces obtained from control and epileptic rats. (**B**–**D**) Quantitative values of total seizure frequency (**B**), total seizure duration (**C**) and seizure severity (**D**) over a 4-day period. Open circles indicate each individual value. Horizontal bars indicate mean value. Error bars indicate SD (* p < 0.05 vs. vehicle (Veh)-treated animals; Mann–Whitney U-test for seizure frequency and seizure severity; Student *t*-test for seizure duration; *n* = 7).

Since conventional anti-epileptic drugs protect CA1 astrocytes from clasmatodendritic degeneration [16], it seems that clasmatodendrosis is relevant to spontaneous seizure activity. Therefore, we evaluated the effect of CDDO-Me on spontaneous seizure activity in epileptic rats over a 4-day period. Under basal (vehicle-treated) conditions, the total seizure frequency was 7.9 ± 1.3/recording session and the total seizure duration was 874.4 ± 227.6 s. The seizure severity (behavioral seizure core) was 3.7 ± 0.5 (Figure 7A–D). CDDO-Me did not affect total seizure frequency (7.6 ± 2.5; Figure 7B). However, the total seizure duration was reduced to 499.6 ± 178.7 s (*t*_(6)_ = 3.27, *p* = 0.02 vs. vehicle, Student *t*-test, *n* = 7, respectively; Figure 7C). The seizure severity (3.4 ± 0.7) was unaffected by CDDO-Me (Figure 7D). These findings indicate that CDDO-ME may decrease seizure duration, but not its frequency and severity, in chronic epileptic rats.

## 4. Discussion

**Figure 8:**
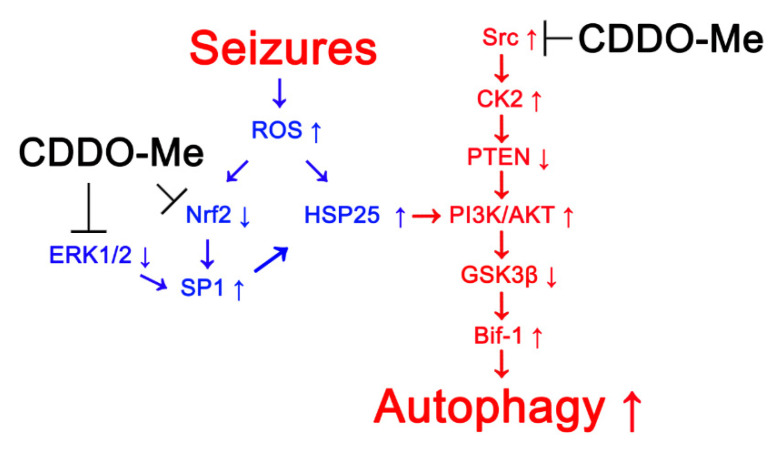
**Schematic depiction representing the effects of CDDO-Me on astroglial autophagy (clasmatodendrosis).** CDDO-Me activates Nrf2-mediated antioxidant defense mechanisms and SP1 suppression, which inhibit further ROS synthesis and HSP25 induction, respectively. In addition, CDDO-Me facilitates ERK1/2-SP1-mediated HSP25 suppression that abrogates PI3K/AKT activation. CDDO-Me also abolishes the PI3K-AKT-Bif-1 signaling pathway by repressing Src-CK2-mediated PTEN inhibition.

The major findings in the present study were that CDDO-Me attenuated HSP25-induced clasmatodendrosis through Nrf2-, ERK1/2-SP1- and Src-CK2-PTEN- PI3K-AKT-GSK3β-Bif-1-mediated signaling pathways in chronic epilepsy rats. In addition, CDDO-Me ameliorated spontaneous seizure duration, but not seizure frequency and behavioral seizure severity (Figure 8).

Clasmatodendrosis was first described by Alzheimer and was postulated to reflect irreversible injury of astrocytes [10]. Clasmatodendrosis is a pathological substrate, linked to white matter hyperintensities, as seen on brain T2-weighted magnetic resonance imaging associated with stroke, Alzheimer’s disease and vascular dementia [53,54]. In addition, influenza-associated encephalopathy [55], traumatic brain injury [56], methamphetamine abuse [57], neuromyelitis optica [58], explosive blasts [59] and osmotic demyelination syndrome [19] induce clasmatodendrosis in the brain gray matter as well as the white matter. Although recent reports have demonstrated that clasmatodendrosis is a corollary of senescence, autophagy, metabolic dysfunction, endoplasmic reticulum (ER) stress or nuclear factor-κB (NFκB) activation [12,18,60,61], the role or the underlying mechanism of this pathological change are not well-defined.

In the present study, clasmatodendritic CA1 astrocytes showed a reduced Nrf2 level in chronic epilepsy rats, and CDDO-Me (an Nrf2 activator) attenuated this astroglial degeneration. Clasmatodendrosis is initiated by acidosis (~pH 5) and energy failure induced by mitochondrial inhibition [62], which are induced by oxidative stress [63,64,65]. This is because ROS reduce glycolysis, leading to intracellular acidosis by the inhibition of Na^+^-H^+^ exchangers and Na^+^-HCO_3_^−^ cotransporters [63], which subsequently impairs the enzymatic steps of glutathione (GSH, an endogenous antioxidant) synthesis [65]. Considering the Nrf2-mediated regulation of γ-glutamyl cysteine ligase and of cystine/glutamate transporters (xCT or SLC7a11), which facilities the up-take of cystine (a GSH precursor) [66], our findings indicate that the dysfunction of Nrf2-mediated antioxidant system may be involved in the initiation of clasmatodendrosis.

Oxidative stress also induces HSP25 (a murine/rodent homologue of human HSP27) that elevates proteasome activity and prevents ROS-induced apoptosis [67,68,69,70]. HSP25 is an inducible HSP that is prominently expressed in astrocytes, and not in neurons, in response to seizure activity [71,72]. In addition, HSP25 is a sensitive and reliable representative marker of the early astroglial energy-consuming events [72,73]. However, impaired clearance of HSP25 reduces astroglial viability via endoplasmic reticulum (ER) stress-induced astroglial autophagy [24,74], although HSP25 facilitates protein folding and the removal of aberrant proteins [75,76]. In previous studies [24,25], sustained HSP25 expression lead to clasmatodendrosis. During this process, the reduced ERK1/2-mediated SP1 phosphorylation induces the prolonged HSP25 upregulation [25], since SP1 phosphorylation inhibits HSP25 transactivation by reducing the SP1 DNA-binding ability [77,78]. In the present study, clasmatodendritic CA1 astrocytes demonstrated HSP25 accumulation with LAMP1 positive vacuoles. Furthermore, CDDO-Me attenuated clasmatodendrosis in CA1 astrocytes concomitant with enhanced ERK1/2 phosphorylation. Since CDDO-Me decreased SP1 expression [79] and increased ERK1/2 activity [35], our findings suggest that CDDO-Me may ameliorate clasmatodendrosis by abrogating prolonged HSP25 upregulation through the repression of SP1 transcription and/or ERK1/2-mediated SP1 phosphorylation, accompanied by Nrf2 activation.

On the other hand, HSP25 binds and forms a new complex with AKT, securing AKT’s natural conformation and enzymatic activity [80]. In addition, HSP25 increases AKT-S473 phosphorylation, which exerts a mechanistic target of rapamycin (mTOR)-independent astroglial autophagy by GSK3β-mediated Bif-1 accumulation [25,81]. Indeed, HSP25 siRNA attenuates clasmatodendrosis by inhibiting the AKT-GSK3β-Bif-1 signaling pathway [24,25,82]. In addition, HSP25 over-expression sustains AKT-S473 phosphorylation by inhibiting the pleckstrin homology domain and leucine-rich repeat protein phosphatase (PHLPP) 1 and 2-binding to AKT (Lee and Kim, 2020). Since HSP25–AKT interaction is not required to promote AKT activation and HSP25 does not interact with PI3K [83,84], it is likely that HSP25 may function as a chaperone to maintain AKT activity rather than an indispensable factor for AKT activation. In the present study, CDDO-Me reduced p-HSP25, p-AKT and Bif-1 levels in the epileptic hippocampus. Regarding the aforementioned role of HSP25 in AKT phosphorylation [82,83,84,85,86], these findings suggest that CDDO-Me-induced HSP25 downregulation may prevent AKT hyper-phosphorylation by diminishing the HSP25 function as a chaperone for AKT.

In the canonical pathway, AKT is dephosphorylated by PTEN [87], which is downregulated by seizure activity [41,42]. Mutation or inactivation of PTEN contributes to seizures in human patients and animal models [88,89,90]. Indeed, PTEN mRNA and its protein expression are downregulated in the rat hippocampus following pentylenetetrazol or kainic acid injection [41,42]. We have also reported that PTEN expression level is lower in the rat epileptic hippocampus than that in the normal hippocampus [39]. In the present study, p-PTEN ratio was similarly observed between the control and the epileptic hippocampus, although PTEN phosphorylation level was lower in the epileptic hippocampus than the normal hippocampus. In addition, CDDO-Me decreased PTEN phosphorylation only in the epileptic hippocampus. Considering that PTEN phosphorylation represents its inactivation [43], these findings indicate that CDDO-Me may increase PTEN activity, and that CDDO-Me may mitigate clasmatodendrosis by facilitating PTEN-mediated AKT inhibition in the epileptic hippocampus.

On the other hand, Src family-Y416 phosphorylation level is significantly decreased in human symptomatic epileptic tissues, as compared to control tissues [91]. Consistent with this previous report, the present study reveals the reduced Src family-Y416 phosphorylation level in the epileptic hippocampus, accompanied by increased AKT phosphorylation. Furthermore, CDDO-Me diminished phosphorylation of Src family-Y416 and CK2-Y255, which are signaling molecules acting as PTEN kinases [44,45,46,47]. Thus, our findings indicate that CDDO-Me may increase PTEN activity by inhibiting Src and CK2 phosphorylation. ERK1/2 also phosphorylates CK2 primarily at T360/S362, subsequently enhancing CK2 activity. Indeed, the level of catenin (a substrate of CK2) phosphorylation correlates with levels of ERK1/2 activity in human glioblastoma [47]. Therefore, it is presumable that CDDO-Me-induced ERK1/2 activation is involved in CK2 activation. However, the present data demonstrate that CK2 T360/S362 phosphorylations were unaltered in the epileptic hippocampus, and unaffected by CDDO-Me. Therefore, it is likely that CK2-Y255 phosphorylation may be involved in PTEN phosphorylation. With respect to CDDO-Me-induced Src and AKT inactivations [51,52], thus, our findings suggest that CDDO-Me may mitigate clasmatodendrosis by regulating the Src-CK2-PTEN-AKT-GSK3β-Bif-1 signaling pathway.

In a previous study, we reported that clasmatodendritic changes might be a consequence of prolonged recurrent seizures induced by hyperexcitability of the temporoammonic path, and not a cause of epileptogenesis. This is because anti-epileptic drugs (valproate, carbamazepine and vigabatrin) prevent clasmatodendrosis in the epileptic hippocampus [16]. However, it could not be excluded the possibility that clasmatodendrosis could affect ictogenesis in the epileptic hippocampus. In the present study, we found that CDDO-Me diminished seizure duration, but not its frequency and severity, in chronic epileptic rats. Astrocytes play an important role in K^+^ buffering [92]. In addition, α-aminoadipic acid (an astroglial toxin) and 4-aminopyridine (a K^+^ channel blocker) synchronize reverberant epileptiform discharges [16,93]. Thus, our findings indicate that clasmatodendrosis may influence seizure duration in the epileptic hippocampus. Indeed, 4,5,6,7-tetrabromotriazole (TBB), a CK2 inhibitor, prevents acute epileptiform discharges [94]. Furthermore, we recently reported that α-amino-3-hydroxy-5-methylisoxazole-4-propionic acid receptor (AMPAR) antagonists decreased CK2 Y255 phosphorylation, but not its T360/S362 phosphorylation levels in chronic epilepsy rats, accompanied by reduced seizure activity [37]. However, they reduced Src-Y416 phosphorylation level, but increased Src-Y527 phosphorylation level [37], unlike CDDO-Me in the present study. Although the direct experimental evidence for a role of clasmatodendrosis in seizure activity is currently limited, it is plausible that clasmatodendrosis may be considered an epiphenomenon affecting seizure duration rather than a primary ictogenic factor in the epileptic hippocampus. In clasmatodendritic astrocytes, aquaporin-4 (AQP4; a water channel) expression is decreased and aggregated in dense peripheral cellular deposits at the edge of rounded or swollen astrocytes in the human brain [54,55]. Similarly, AQP4 expression is negligible in clasmatodendritic CA1 astrocytes in the hippocampus of chronic epileptic rats [17]. Interestingly, astrocytes lacking AQP4 increase seizure duration, since AQP4 deletion delays clearance of K^+^ from extracellular space [95]. With respect to these previous reports, it is likely that clasmatodendritic astrocytes may lead to the slow clearance of K^+^ and water from extracellular space in the ictal stage, which could be involved in the duration and propagation of synchronous discharges in the epileptic hippocampus. Further studies are needed to elucidate the underlying mechanisms concerning the role of clasmatodendrosis in ictogenesis.

## 5. Conclusions

In the present study, we demonstrated, for the first time, that CDDO-Me ameliorated HSP25-induced astroglial autophagy via Nrf2-, ERK1/2-SP1- and Src-CK2-PTEN-PI3K-AKT-GSK3β-Bif-1-mediated signaling pathways in chronic epilepsy rats. In addition, CDDO-Me shortened seizure duration. Therefore, our findings suggest that autophagic astroglial degeneration may play an important role in the maintenance of spontaneous seizure activity, which could be one of the therapeutic targets for TLE medications.

## Data Availability

Not applicable.

## Supplementary Materials

The following are available online at https://www.mdpi.com/article/10.3390/antiox10050655/s1, Table S1: Primary antibodies used in the present study, Figure S1: Representative photos of negative control test, Figure S2: Representative full-gel images of Western blots in Figure 1E and Figure 3A, Figure S3: Representative full-gel images of Western blots in Figure 4A, Figure S4: Representative full-gel images of Western blots in Figure 5A and Figure S5: Representative full-gel images of Western blots in Figure 6A.

## Abbreviations

AMPAR, α-amino-3-hydroxy-5-methylisoxazole-4-propionic acid receptor; ANOVA, analysis of variance; AQP4, aquaporin-4,; ARE, antioxidant-response element; Bif-1, bax-interacting factor 1; CDDO-Me, 2-cyano-3,12-dioxo-oleana-1,9(11)-dien-28-oic acid methyl ester; CK2, casein kinase 2; Cul3, cullin-3; DAPI, 4′,6-diamidino-2-phenylindole; EEG, electroencephalogram; ER, endoplasmic reticulum; ERK1/2, extracellular signal-related kinases 1/2; GFAP, glial fibrillary acidic protein; GSH, glutathione; GSK3β, glycogen synthase kinase 3β; GSTs, glutathione S-transferases; HSP25, heat shock protein 25; i.p., intraperitoneal; Keap1, Kelch-like erythroid cell-derived protein with CNC homology (ECH)-associated protein 1; LAMP1, lysosomal-associated membrane protein 1; mTOR, mechanistic target of rapamycin; Nrf2, nuclear factor-erythroid 2-related factor 2; PI3K, phosphatidylinositol-3-kinase; PPLPP, pleckstrin homology domain and leucine-rich repeat protein phosphatase; PTEN, phosphatase and tensin homolog deleted on chromosome 10; ROS, reactive oxygen species; SP1, specificity protein 1; TBB, 4,5,6,7-tetrabromotriazole; TLE, temporal lobe epilepsy; TUNEL, terminal deoxynucleotidyl transferase dUTP nick-end labeling; UPS, ubiquitin proteasome system.
