# Supplementary material for: CDDO-Me Attenuates Astroglial Autophagy via Nrf2-, ERK1/2-SP1- and Src-CK2-PTEN-PI3K/AKT-Mediated Signaling Pathways in the Hippocampus of Chronic Epilepsy Rats
Source: Antioxidants (Basel). 2021 Apr 23;10(5):655. doi: 10.3390/antiox10050655 (PMC8145743; doi:10.3390/antiox10050655)
Supplement: Supplementary file 1 [file antioxidants-10-00655-s001.zip › antioxidants-1148773-PE supp update.pdf]

Supplementary information

CDDO-Me Attenuates Astroglial Autophagy via Nrf2-, ERK1/2-SP1- and Src-CK2-PTEN-PI3K/AKT-Mediated Signaling Pathways in the Hippocampus of Chronic Epilepsy Rats

Ji-Eun Kim[1,2]*and Tae-Cheon Kang[1,2]*

1 Department of Anatomy and Neurobiology, College of Medicine, Hallym University, Chuncheon 24252, South Korea

2 Institute of Epilepsy Research, College of Medicine, Hallym University, Chuncheon 24252, South Korea

* Correspondence to: J. -E. Kim, Department of Anatomy and Neurobiology, College of Medicine, Hallym University, Chuncheon, Kangwon-Do 24252, South Korea; Tel: +82-33-248-2522; Fax: +82-33-248-2525; E-mail: jieunkim@hallym.ac.kr; and T. -C. Kang, Department of Anatomy and Neurobiology, College of Medicine, Hallym University, Chuncheon, Kangwon-Do 24252, South Korea; Tel: +82-33-248-2524; Fax: +82-33-248-2525; E-mail: tckang@hallym.ac.kr

Supplementary Table S1. Primary antibodies used in the present study.**Antibody**

Antibody	Host	Manufacturer (catalog number)	Dilution used
AKT	Rabbit	Cell signaling (#9272)	1:1,000 (WB)<br>1:50 (IP)
Bif-1	Rabbit	Cell signaling (#4427)	1:1,000 (WB)
CK2	Mouse	Millipore (#05-1431)	1:1,000 (WB)
ERK1/2	Rabbit	Biorbyt (orb160960)	1:1,000 (WB)
GFAP	Mouse	Millipore (mab3402)	1:4,000 (IF)
HSP25	Rabbit	Enzo (ADI-SPA-801)	1:1,000 (WB)<br>1:500 (IF)
LAMP1	Rabbit	Lifespan (LS-B580)	1:1,000 (WB)<br>1:200 (IF)
Nrf2	Mouse	Abcam (ab89443)	1:1,000 (WB)<br>1:100 (IF)
p-AKT-S473	Rabbit	Cell signaling (#4060)	1:1,000 (WB)
p-CK2-Y255	Rabbit	Invitrogen (#PA5-38831)	1:1,000 (WB)
p-CK2-T360/S362	Rabbit	Abcam (ab119410)	1:1,000 (WB)
p-ERK1/2	Rabbit	Bioss (bs-3330R)	1:1,000 (WB)
p-PTEN	Rabbit	Cell signaling (#9549)	1:1,000 (WB)
p-Src-Y416	Rabbit	Cell signaling (#6943)	1:1,000 (WB)
PTEN	Rabbit	Abcam (ab32199)	1:10,000 (WB)
Src	Rabbit	Cell signaling (#2108)	1:1,000 (WB)
β-actin	Mouse	Sigma (A5316)	1:5,000 (WB)

IF, Immunofluorescence; WB, Western blot.

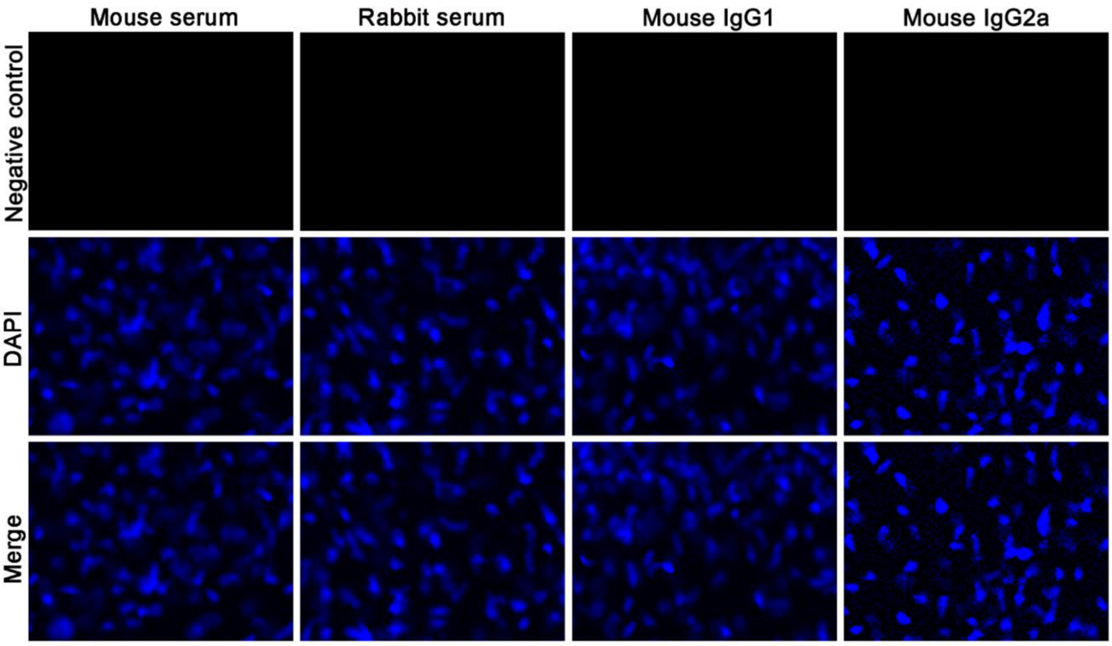

Supplementary Figure S1. Representative photos of negative control test.

Fig. 1E

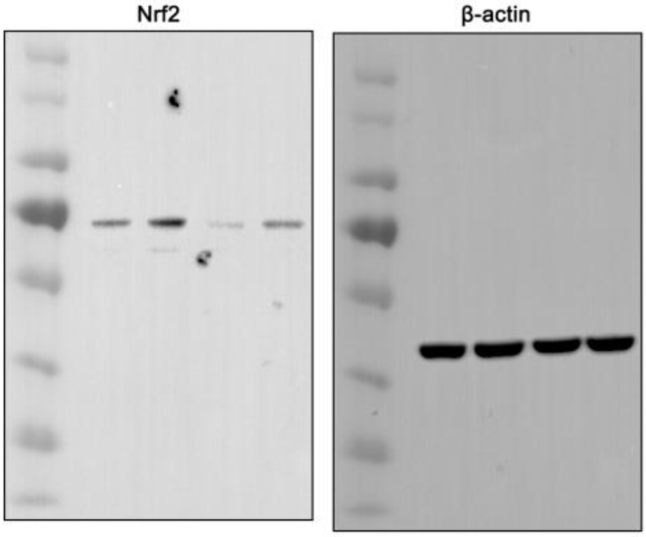

Fig. 3A

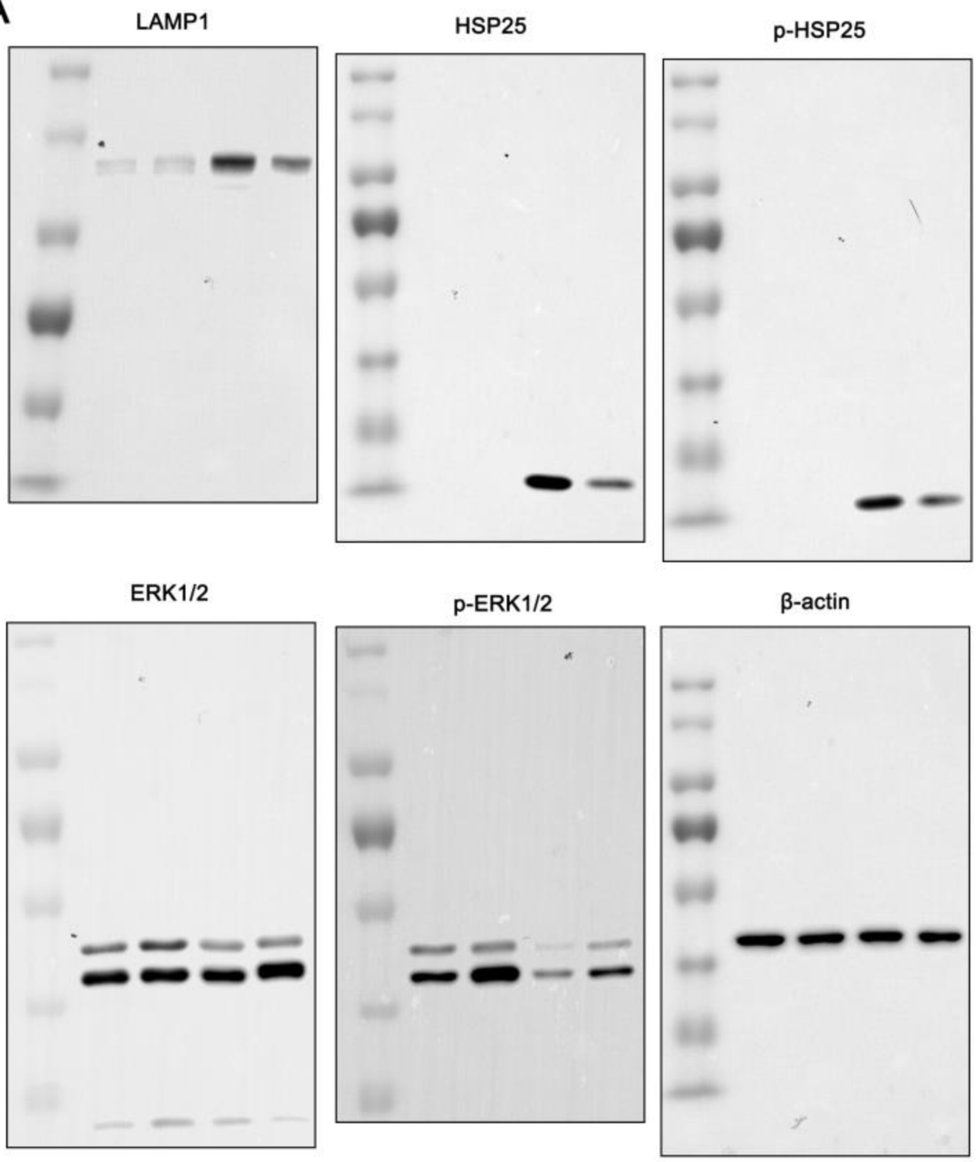

Supplementary Figure S2.Representative full-gel images of Western blots in Figure 1Eand 3A.

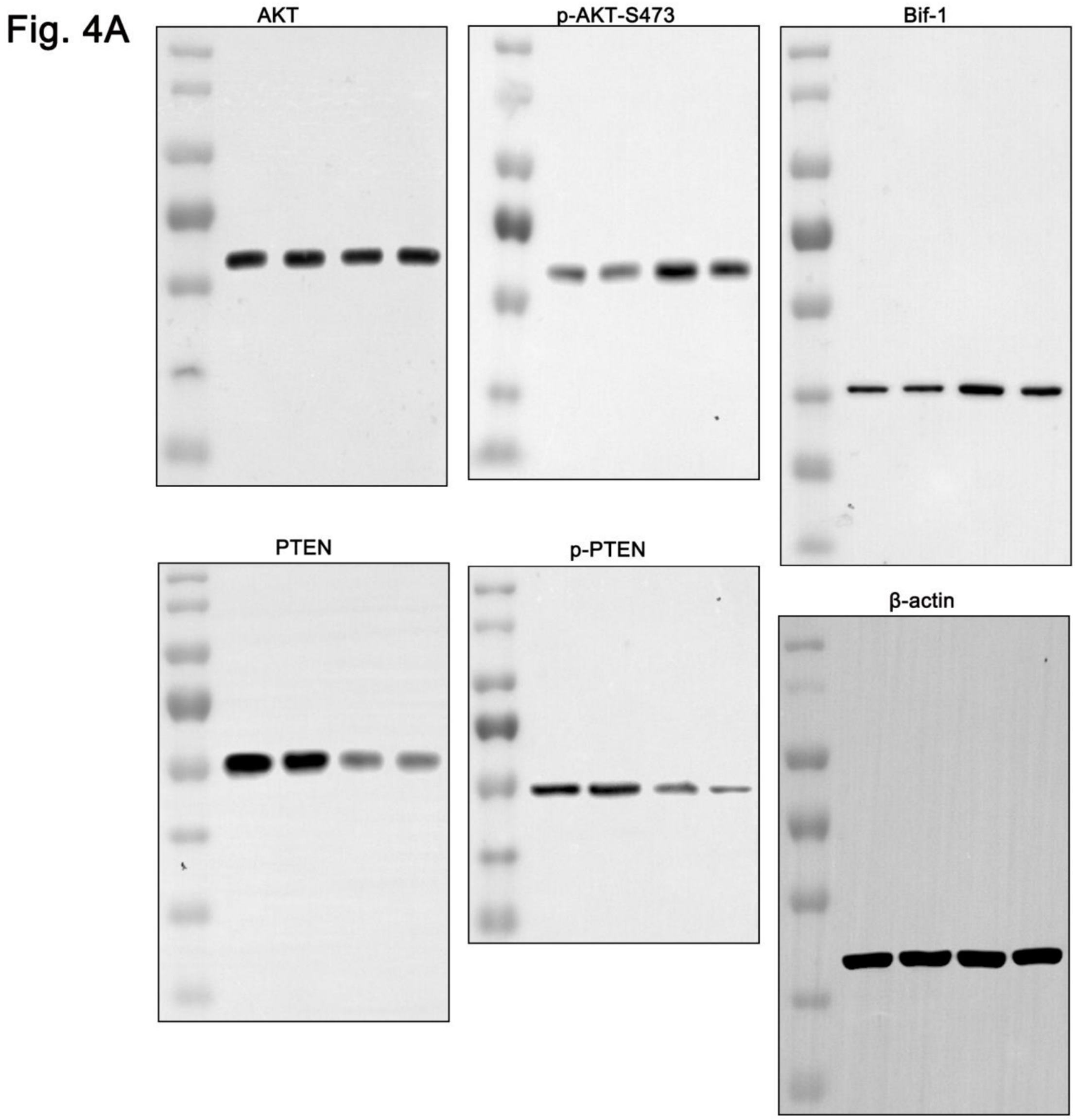

Supplementary Figure S3. Representative full-gel images of Western blots in Figure 4A.

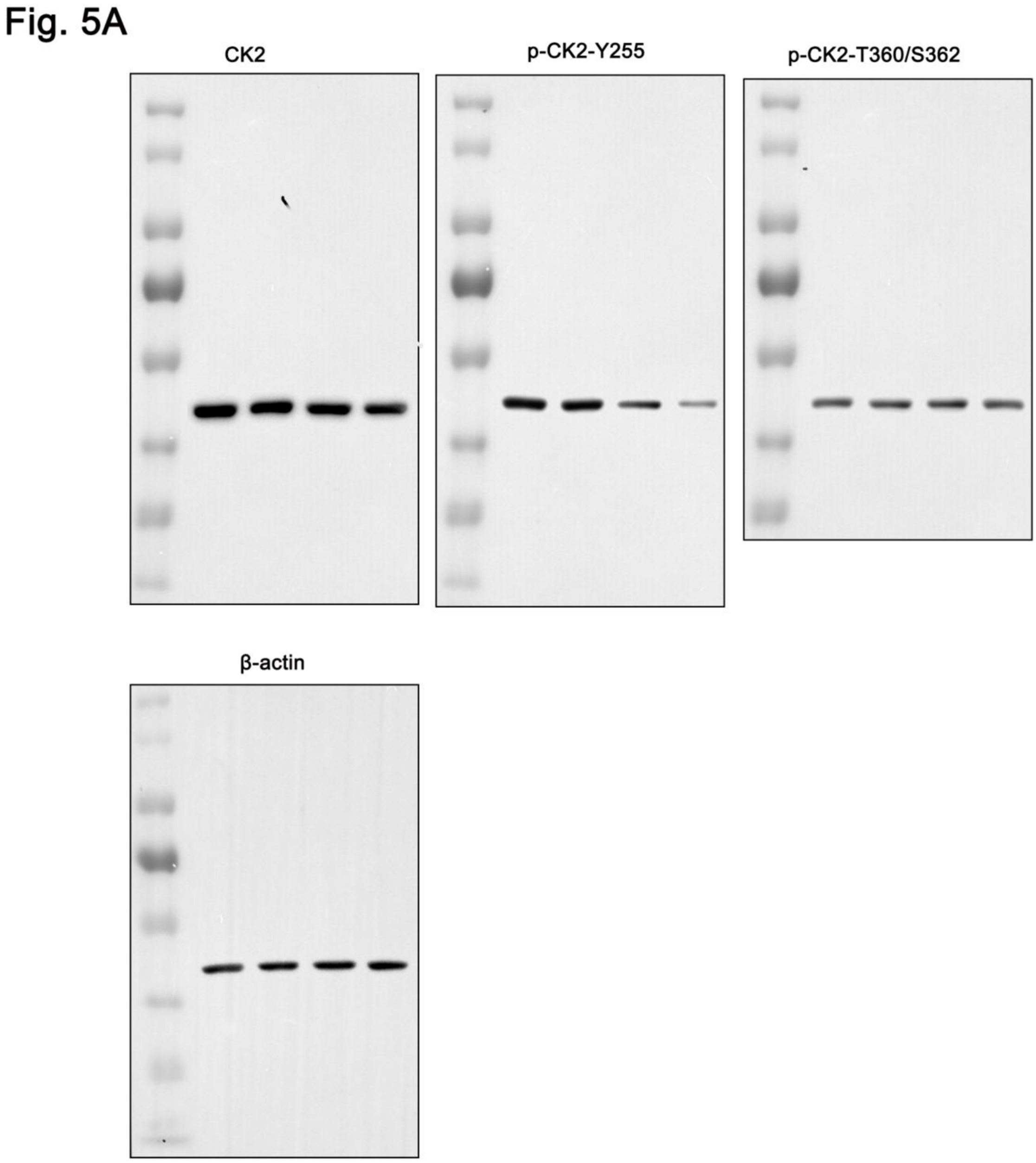

Supplementary Figure S4. Representative full-gel images of Western blots in Figure 5A.

Fig. 6A

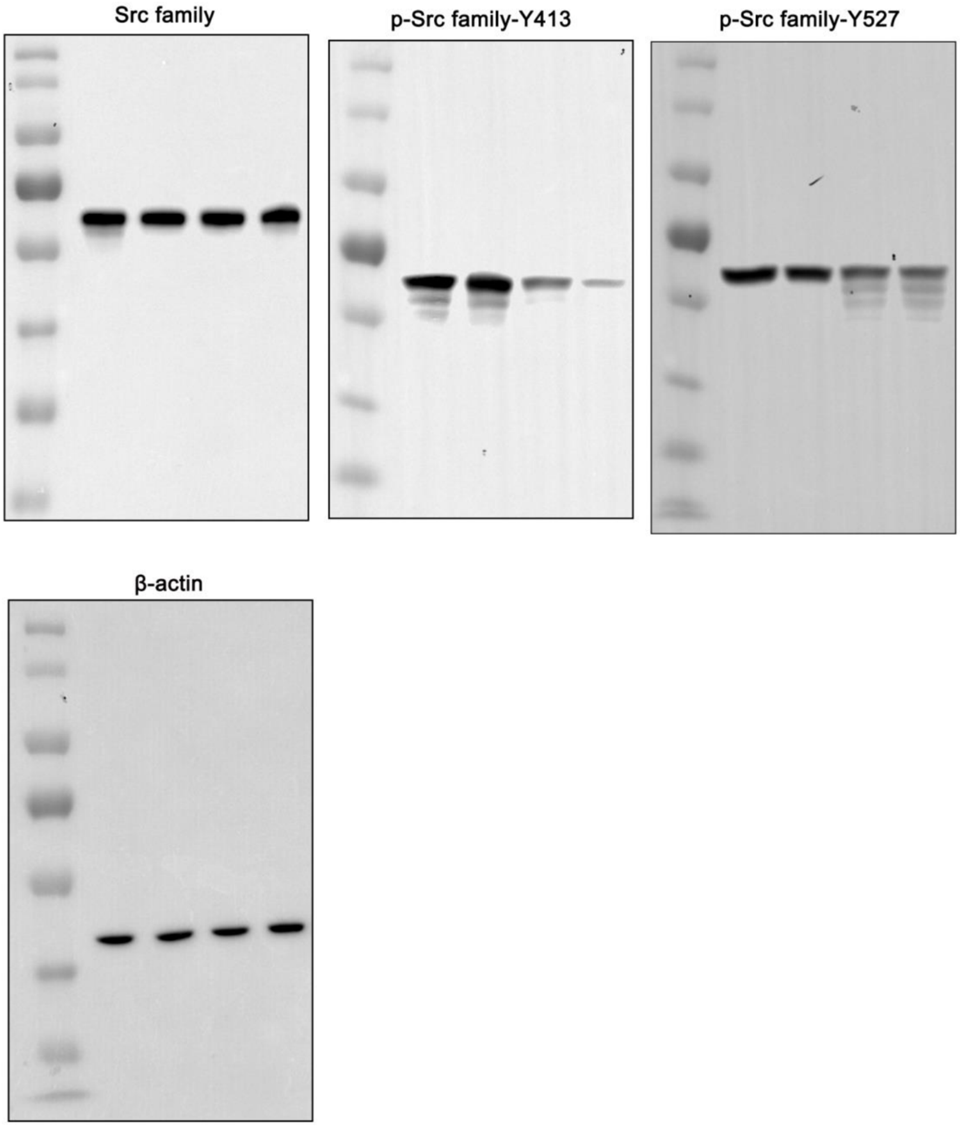

Supplementary Figure S5. Representative full-gel images of Western blots in Figure 6A
